# Impact of Hospitalization in an Endocrinology Department on Vaccination Coverage in People Living with Diabetes: A Real-Life Study

**DOI:** 10.3390/medicina58020219

**Published:** 2022-02-01

**Authors:** Laura Lohan, Charlène Cool, Loriane Viault, Philippe Cestac, Eric Renard, Florence Galtier, Maxime Villiet, Antoine Avignon, Ariane Sultan, Cyril Breuker

**Affiliations:** 1 Clinical Pharmacy Department, CHU Montpellier, University of Montpellier, 34295 Montpellier, France; l-lohan_descamps@chu-montpellier.fr (L.L.); loriane.viault@aphp.fr (L.V.); m-villiet@chu-montpellier.fr (M.V.); 2 Phymedexp, University of Montpellier, INSERM, CNRS, CHRU de Montpellier, 34295 Montpellier, France; a-sultan@chu-montpellier.fr; 3 Department of Pharmacy, Toulouse University Hospital, 31059 Toulouse, France; cool.c@chu-toulouse.fr (C.C.); cestac.p@chu-toulouse.fr (P.C.); 4 Centre for Epidemiology and Population Health Research (CERPOP), UMR 1027, INSERM, University of Toulouse (UPS), 31059 Toulouse, France; 5 Endocrinology-Diabetology-Nutrition Department, University of Montpellier, 34295 Montpellier, France; e-renard@chu-montpellier.fr (E.R.); f-galtier@chu-montpellier.fr (F.G.); a-avignon@chu-montpellier.fr (A.A.)

**Keywords:** people living with diabetes, vaccination coverage, hospitalization, clinical pharmacist

## Abstract

*Background and Objectives*: Vaccination coverage is suboptimal in people living with diabetes. The objectives of this study were to determine the impact of hospitalization on vaccination coverage and the variables associated with vaccination during hospital stay. *Materials and Methods*: This observational study was conducted from May 2019 to December 2019 in the Endocrinology-Nutrition-Diabetes Department of the University Hospital of Montpellier, France. This department encompasses three medical units, two of which have a full-time clinical pharmacist involved in the multidisciplinary management of patients. All adult diabetic patients who completed a questionnaire about vaccines were prospectively included by a clinical pharmacist and followed until department discharge. Coverage at the time of admission for the tetanus, diphtheria, pertussis (Tdap), pneumococcal, influenza, and herpes zoster vaccines was assessed from patient interviews and/or contact with the general practitioner and/or with the community pharmacist. Multivariable logistic regression analysis was performed to identify the factors associated with a vaccination update during the hospital stay. *Results*: A total of 222 patients were included (mean age: 59.4 years, 68.5% type 2 diabetes). Vaccination coverage increased by 26.7% (47.3% to 59.9%), 188.0% (10.8% to 31.1%) and 8.9% (45.9% to 50.0%), respectively, for the Tdap, pneumococcal and influenza vaccines during hospital stay. Female sex, admission to a diabetes care unit with a full-time pharmacist, favorable feelings about vaccination, unknown immunization coverage for pneumococcal vaccines, and evaluation and recording of vaccine coverage at admission in the patient medical records were associated with at least one vaccination during hospital stay. *Conclusions*: Our real-life study highlights that hospitalization and multidisciplinary management (i.e., physician-pharmacist) may be key points in the diabetes care pathway to improve vaccination coverage, especially for patients with advanced diabetes and comorbidities.

## 1. Introduction

Managing people with diabetes is largely based on a prevention approach, with vaccination as an essential component. Infections are frequent complications that seriously affect the quality of life and life expectancy of this population. Indeed, a higher risk of infection than in the general population has been demonstrated, and diabetes is classified as a moderate acquired immune deficiency [1,2,3]. Influenza and pneumococcal diseases are common in people with diabetes, who show high morbi-mortality in terms of risk of hospitalization and death [4]. In addition to these pulmonary infections, diabetes is also a risk factor for tetanus, zona, hepatitis B infections, and invasive meningococcal disease [5,6,7].

Yet, these infections can be prevented by vaccination, which has been recommended by various national health services and diabetology societies [5,8,9,10,11,12]. The recommendations specify that certain vaccinations should be mandatory for the general adult population (diphtheria, pertussis) and that others should be given to people living with diabetes (influenza, pneumococcal, hepatitis B, meningococcal, COVID-19, measles, mumps and rubella, and varicella) or those between 65 and 74 years old, independently of diabetes (herpes zoster) [4,13,14,15,16,17,18,19,20]. Despite these recommendations, a lack of vaccination coverage has been observed. For example, seasonal influenza vaccine coverage among people with diabetes ranges from 32% to 85% depending on the country, age group, and season [21]. There are only limited data on the pneumococcal vaccine coverage of diabetic patients, with the estimations ranging between 20% and 30% [14]. The obstacles to vaccination are multiple and of various origins [22,23,24], which suggests that the input and skills of all health professionals across primary, community, and specialist care are essential to provide high quality care for people with diabetes. Thus, pharmacists, in addition to physicians and nurses, should be involved in managing immunization coverage, particularly in at-risk populations. Indeed, when pharmacists are involved in immunization efforts, whether as educators, facilitators, or administrators of vaccines, they help optimize vaccine coverage for influenza, pneumococcal infection and, more recently, COVID-19 [25,26,27,28].

Hospitalization may be an appropriate moment for assessing immunization coverage and recommending vaccinations. Indeed, clinical pharmacy activities, such as medication reconciliation and medication reviews, provide clear opportunities for patient vaccination assessment so that the medical team can then recommend vaccination updates to the patients during their hospital stay.

The main objective of our study was to assess whether the hospitalization of diabetic patients offered an opportunity for vaccination updates. The secondary objective was to assess (i) the vaccination coverage of hospitalized diabetic patients with comorbidities and (ii) the contribution of the pharmaceutical team in improving coverage, if needed.

## 2. Materials and Methods

### 2.1. Study Design and Participants

From May 2019 to December 2019, we conducted an observational study in the Endocrinology-Nutrition-Diabetes Department of the University Hospital of Montpellier, France. This department encompasses three medical units, including two full-time inpatient units (units 1 and 2) and one weekday hospitalization unit. In unit 1, and the weekday unit, a full-time clinical pharmacist is present and participates in the multidisciplinary management of patients. For full-time unit 2, the clinical pharmacist is only present during patient inclusion and does not participate in patient management.

All patients over 18 years old treated for type 1 or 2 diabetes were eligible for this study. Within 24 h of admission to the department, a clinical pharmacist asked each patient to complete a questionnaire on personal feelings and knowledge about immunization coverage of mandatory and recommended vaccines according to the French Health Ministry [13]. Patients who agreed to complete the questionnaire were prospectively included in the study and followed until department discharge (*n* = 222). They received standard clinical care and the study followed the principles of the Declaration of Helsinki and the ethical standards in France. The study protocol was approved by the Institutional Review Board of our university hospital (Comité Local d’Ethique Recherche, n°2019_IRB-MTP_12-10) and was registered on ClinicalTrials.gov (NCT04391088).

### 2.2. Interventions

If available, a member of the clinical pharmaceutical team (senior pharmacist or resident) carried out the medication reconciliation process within 24 h of admission or on the first working day following weekend admission in the three units. The process followed a validated protocol [29] and included the Best Possible Medication History (BPMH), defined as the most comprehensive list of all medications taken by the patient, including prescription drugs and self-medication. The BPMH was usually obtained through a detailed and structured patient or family interview and contact with the community pharmacy, general practitioner, or nurse. A patient questionnaire, requiring approximately 10 min to complete, was specifically developed for this study, and was proposed to the patient during the medication reconciliation.

The questionnaire included questions on (i) knowledge about the mandatory (tetanus, diphtheria, pertussis: Tdap vaccine) and recommended (influenza and pneumococcal vaccines and, if >65 years old, the herpes zoster vaccine) vaccinations for people living with diabetes, according to French recommendations [13]; (ii) the possession and updating of a vaccination record booklet; and (iii) feelings about vaccines (favorable, unfavorable, mixed or no opinion).

**Figure 1:**
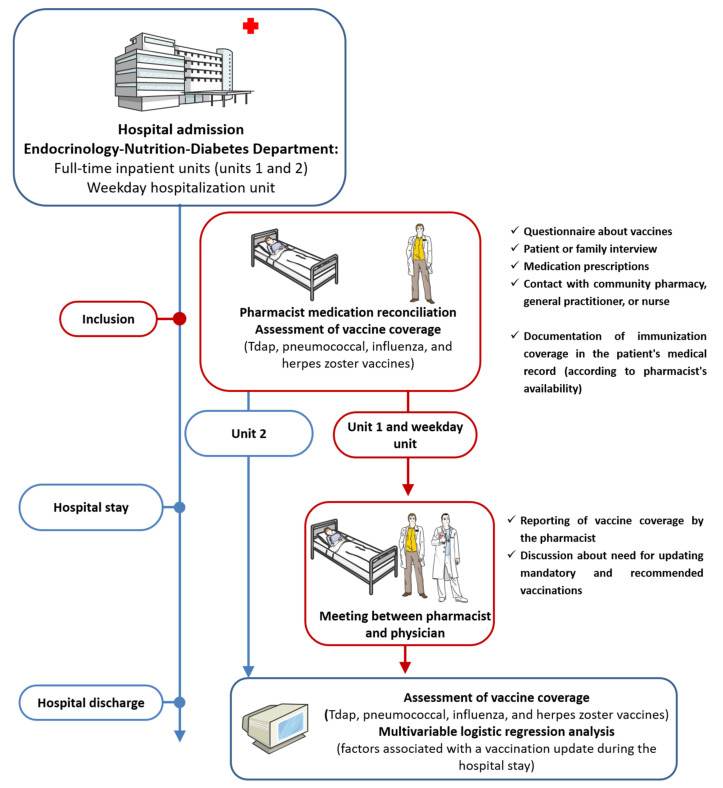
Summary of the interventions.

For the three units, vaccination coverage (up to date, not up to date, unknown) for mandatory and recommended vaccines (Tdap, influenza and pneumococcal vaccines, and herpes zoster vaccine, if applicable) was assessed by a clinical pharmacist at hospital admission from patient interviews and/or contact with the general practitioner and/or contact with the community pharmacist. For unit 1 and the weekday unit, this assessment was followed by a meeting with the physician responsible of the patient, at which time the pharmacist reported the findings. At this meeting, the need for updating mandatory and recommended vaccinations was discussed. The pharmaceutical team could document vaccine coverage in the patient’s medical record for the three units. Mandatory and recommended vaccines were again assessed at hospital discharge, with specific attention paid to any vaccinations performed during hospitalization (Figure 1).

### 2.3. Data Collection

In addition to the data provided by the questionnaire, demographic, clinical, therapeutic, and biological data were prospectively collected from the medical records: age, sex, type and duration of diabetes, body mass index, HbA1c, and treatment.

### 2.4. Statistical Analysis

Patient characteristics and questionnaire responses are expressed as number and percentages for categorical variables and mean ± standard deviation (SD) for quantitative variables. For comparative purposes, results are presented for patients up to date with the Tdap, influenza or pneumococcal vaccines or not, and for patients with or without vaccination during the hospital stay.

Comparisons between categorical variables were performed using chi-square tests or Fisher’s exact tests (for expected values < 5). Student’s *t*-tests, Fisher’s exact tests, or the analysis of variance were used for continuous variables.

Multivariable logistic regression analyses were performed to identify which factors were associated (1) with vaccination coverage for the Tdap, influenza, and pneumococcal vaccines at hospital admission, and (2) vaccinations brought up to date during the hospital stay.

For each analysis, all independent variables with a *p*-value < 0.25 in the bivariate analysis were simultaneously introduced in the models (full models). We used a manual backward stepwise regression procedure, with a significance level of 0.05 to exclude variables from each full model. All variables were included in the multivariable models after testing the interactions between covariates (with a significance level of 0.05 [30]). All models were adjusted for potential confounders. Goodness-of-fit for the logistic regression models was considered acceptable if the Hosmer–Lemeshow test had a *p*-value > 0.05 [31]. All analyses were conducted using SAS 9.4^TM^ software (SAS Institute, Inc., Cary, NC, USA).

## 3. Results

### 3.1. Patient Characteristics

**Figure 2:**
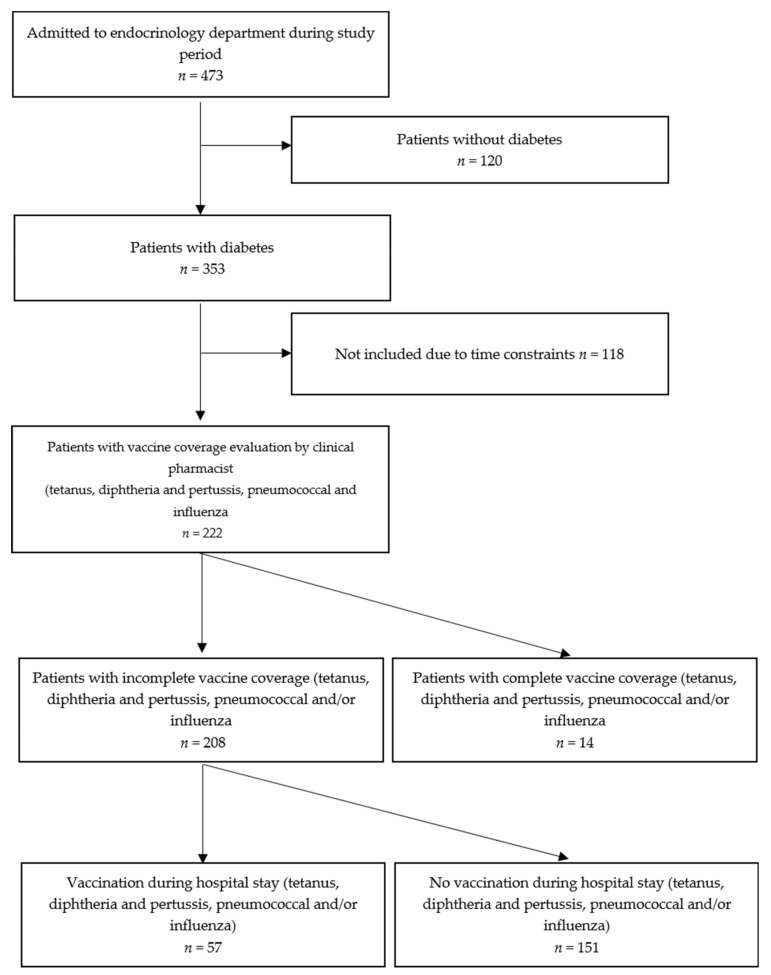
Flowchart of the study population.

During the study period, 473 patients were admitted into one of the three medical units, including 120 patients without diabetes and 118 who could not be included in the study due to time constraints. We examined all the data on the patients with diabetes, and vaccination coverage was evaluated by a clinical pharmacist (*n* = 222) (Figure 2). The population is described in Supplementary Table S1. The mean age was 59.4 ± 15.2 years, 59.0% were men, 68.5% had type 2 diabetes, the mean number of medications was 7.9 ± 4.3, 68.5% were on insulin, and the mean diabetes duration was 9.0 ± 13.9 years.

### 3.2. Vaccination during Hospitalization

Respectively, 105 (47.3%), 24 (10.8%), and 102 (45.9%) patients were up to date for the Tdap, pneumococcal, and influenza vaccines. Immunization coverage could not be established with certainty and was classified as “unknown” for the Tdap, pneumococcal, and influenza vaccines for, respectively, 44 (19.8%), 24 (10.8%), and 2 (0.9%) patients. A total of 10.5% (70) of the 666 evaluated vaccine coverages had unknown status. None of the 79 patients over 65 years of age were vaccinated against the herpes zoster virus. In total, 179 (80.6%) patients had known immunization coverage for all three vaccines (up to date or not up to date) and 14 (6.3%) patients were up to date for the three vaccines (Supplementary Table S1). Vaccination coverage established at hospital admission by the pharmacist was documented in the medical records of 183 patients (82.4%): 53 (89.8%) patients in full-time unit 1, 63 (73.3%) patients in full-time unit 2, and 67 (87.0%) patients in the weekday unit.

In total, 208 patients had incomplete vaccination coverage at admission for at least one of the three vaccines (not up to date or unknown status) (Table 1).

A total of 57 (27.4%) patients were vaccinated during their hospital stay, and 28 (23.9% of not-up-to-date or unknown status, 28/117), 45 (22.7% of not-up-to-date or unknown status, 45/198) and 9 (20.5% of not-up-to-date or unknown status end according to winter season, 9/44) received vaccinations with Tdap, pneumococcal and influenza vaccines. Patients whose vaccination coverage was recorded by the pharmacist in their medical record received significantly more vaccines during their stay (96.5% vs 76.8%, *p* = 0.0009) (Table 1).

Therefore, vaccination coverage increased by 26.7% (47.3% to 59.9%), 188.0% (10.8% to 31.1%) and 8.9% (45.9% to 50.0%), respectively, for the Tdap, pneumococcal and influenza vaccines during hospital stay (Table 2). The increase in vaccination coverage for the three vaccines was greater in the units that included a clinical pharmacist: Tdap (42.8% vs. 4.5%), pneumococcal (313.6% vs. 10.4%) and influenza (12.0% vs. 2.9%).

In 94.2% of the cases, the vaccinations during hospital stay were recorded in the medical discharge report.

### 3.3. Knowledge and Feeling about Vaccines

In our study population, 171 (77.0%) and 8 (3.6%) patients, respectively, were aware of the mandatory (Tdap vaccine) and the 2 recommended vaccines (pneumococcal and influenza vaccines) (Table 3). Eighty-three patients could cite influenza and nine could cite pneumococcal as the recommended vaccines. Concerning patient feelings about vaccines, 19 (8.6%) were against them and 54 (24.3%) had mixed feelings. The main declared reasons were the poor effectiveness of the vaccine (27%), side effects (30.2%), and fear (11.1%).

### 3.4. Factors Associated with Vaccination Coverage at Hospital Admission and Vaccination during Hospitalization

Multivariate analysis highlighted that female sex (OR IC95%, 0.14 (0.02–0.90), *p* = 0.04) and the reason for hospitalization (insulin pump installation OR IC95%, 0.01 (0.001–0.28), *p* = 0.02) were associated with a lack of immunization coverage for Tdap, whereas advanced diabetes (diabetes duration ≥10 years) was associated with up-to-date vaccination coverage (OR IC95%, 19.7 (1.64–235.5)). For the influenza vaccine, age (OR IC95%, 1.04 (1.01–1.06), *p* = 0.004), number of medications taken (OR IC95%, 1.09 (1.01–1.18), *p* = 0.03), and duration of diabetes (OR IC95%, 1.03 (1.00–1.05), *p* = 0.02) were found to be associated with vaccine coverage. No variables of interest were statistically associated with pneumococcal vaccine coverage in our analysis.

Factors associated with at least one vaccination during hospitalization are described in Table 4. Female sex, diabetes care unit with a team of clinical pharmacists (full-time inpatient unit 1 and weekday unit), feelings about vaccination, immunization coverage with the pneumococcal vaccine, and documentation in the medical record of the pharmacist’s assessment of the vaccination coverage on hospital admission were associated with at least one vaccination during hospital stay.

## 4. Discussion

Our study demonstrates that hospital stay in an endocrinology department resulted in improved vaccination coverage in high-risk diabetic patients. In addition, our results confirmed that vaccination coverage is insufficient, as already demonstrated, and extended the results to those with long-standing diabetes and associated comorbidities. Finally, the pharmacist’s inclusion of the vaccination coverage status in the patient medical records was associated with an improvement in vaccination coverage.

These results are particularly important, given the observation of insufficient vaccine coverage, as already described. In addition, we included hospitalized patients with long-standing diabetes and complications/comorbidities, who are particularly at risk of infection. Our study shows that hospitalization can be a key moment to improve vaccination coverage. We identified the variables associated with vaccination coverage that help in targeting patients (reason for hospitalization, lack of knowledge about recommended vaccines, age, number of medications and duration of diabetes) and the variables associated with vaccination during hospitalization. For example, female sex, feelings about vaccination, pneumococcal vaccination coverage, a medical unit that included a clinical pharmacist, and documentation in the medical record of the pharmacist’s assessment of the vaccination coverage on hospital admission were associated with at least one vaccination during hospital stay. Indeed, these variables indicate that hospitalization and the presence of a pharmacist were able to overcome some of the obstacles to vaccination reported by general practitioners, such as the lack of time to determine coverage status and limited resources. We were indeed able to vaccinate many of the patients favorable to vaccination. However, improvement is still needed, as 64 patients in our study were in favor of vaccination but were not vaccinated, indicating that there were other barriers to vaccination during their stays. Last, vaccination coverage increased between 8.9% to 188.0%, depending on the vaccine, during hospital stay, with this rate higher in units including a full-time clinical pharmacist (12.0% to 313.6%). Blanchi et al. also highlighted the impact of hospitalization on Tdap vaccination coverage in people over 65 years of age. In this randomized interventional study, Tdap vaccination coverage increased by 43.8% (56.2% to 80.8%) in the interventional arm versus 6.3% (38.1% to 40.5%) in the control arm [32]. Our results were similar, especially when we compared units with and without pharmacists (+42.8%, 44.8 to 64.0% vs. +4.5%, 51.2% to 53.5%).

Difficulty in evaluating immunization coverage is a deterrent to vaccination and an obstacle in keeping vaccinations up to date [32]. In our study, we found a lower rate of unknown vaccine status compared to the study of the French-speaking Diabetes Society for influenza (0.9% vs. 3.5%) and pneumococcal (10.8% vs. 32.6%) and a slightly higher rate for Tdap (19.8% vs. 14.9%), although we had a much lower percentage of patients with a vaccination booklet (26.6% vs. 52.0%) [33]. When we compared the overall rate of unknown vaccine coverage for these three vaccines, we found a rate lower than that of the French-speaking Diabetes Society (10.5% vs. 17%) [33]. This difference can be explained by the methodology used, as the Diabetes Society study used only a patient survey, whereas in our study we had several sources of information (patient, general practitioner, and community pharmacist) and we involved a clinical pharmacist.

Regarding how diabetic patients feel about vaccinations, we found that only half the patients were in favor of them (50.9%). This result is comparable to the findings of a study conducted by the “Vaccination of the Diabetic Person” Working Group of the French-speaking Diabetes Society on an ambulatory population of diabetic patients [33]. In addition, a survey of 140 countries and 140,000 people highlighted the significant skepticism of the French population regarding vaccination, with one in three individuals considering vaccines as unsafe, which is the highest percentage worldwide [34]. In 2016, Larson et al. also found the French population to be the most skeptical (45%) about vaccination [35]. Vaccine skepticism in France is not new, and it increased after the controversial influenza pandemic vaccination campaign in 2009 [36]. In addition to this skepticism regarding vaccination, there is poor knowledge about the recommended vaccines. This may be due to the insufficient attention that physicians give to the recommendations during medical consultations, as noted by the French-speaking Diabetes Society [33], and to a lesser extent it may be due to the vaccine hesitancy of some health professionals. In one study, Verger et al. found that 16% to 43% of 1712 French general practitioners on a randomly selected national panel only sometimes or never recommended at least one specific vaccine to their target patients [22]. In this cross-sectional observational study, the general practitioners who recommended vaccines were the physicians most comfortable explaining the benefits and risks to patients and those who trusted official sources of information. Conversely, physicians who rarely recommended vaccines were those who feared adverse effects and had doubts about their usefulness [22]. Healthcare providers remain the most trusted advisors and influencers of vaccination decisions, but they must now deal with time constraints due to the increasing number of hesitant patients and they may need more information and training to answer the many questions about vaccination [37].

The COVID-19 pandemic and the risk profile of diabetic patients are prompting health professionals and authorities to redouble their efforts to improve vaccination coverage against pulmonary infectious agents, particularly influenza [38,39]. Hospital stays should be considered as opportunities to improve vaccine coverage in diabetic patients.

In addition, the recommendations must adapt to new scientific data. It has recently been shown that type 1 diabetes mellitus showed significant association with hospitalization for invasive meningococcal disease in a French national public health insurance database [7]. The addition of meningococcal vaccination in the vaccine recommendation for type 1 diabetics could be an evolution of the French vaccination calendar. This is the case in some countries, such as Italy [16,18].

Some limitations of our study should be noted. This was a monocentric real-life study that enrolled a modest number of participants, although we were able to detect significant differences and relevant variables. In addition, only inpatients were included in this study, making it difficult to compare with outpatients. Moreover, our observational design, without a control arm, did not allow us to draw any conclusions about the specific role of pharmacists in improving quality of care.

Despite these limitations, the key strengths of the study include (i) the rigorous assessment of immunization coverage, (ii) the assessment of patients’ feelings about vaccination, and (iii) the identification of variables of interest

## 5. Conclusions

Our real-life study highlights that hospital stays and multidisciplinary physician-pharmacist management may be key points in the diabetes care pathway to improve vaccination coverage, especially for patients with advanced diabetes and comorbidities. The evaluation of the vaccination coverage and patient feelings about vaccination are important factors to improve vaccination. Last, the involvement of a pharmacist can help improve the status of vaccination coverage, and this new activity is compatible with pharmaceutical activities, such as medication reconciliation, which helps reduce medication errors, detection of adverse events, and promotion of compliance with management recommendations [40,41,42,43].

## Figures and Tables

**Table 1:** Characteristics of population with incomplete vaccine coverage and vaccination during hospital stay.

	Incomplete Immunization Coverage at Admission	Vaccination during Hospital Stay		
		NO	YES	*p*-Value
n	208	151 (72.6)	57 (27.4)	
Sex, male	119 (57.2)	91 (60.3)	28 (49.1)	0.16
Age (years), mean (sd)	59.5 ± 15.0	59.1 ± 15.8	60.3 ± 12.9	0.57
Type 2 diabetes	145 (69.7)	102 (67.6)	43 (75.4)	0.31
Length of stay, days	8.6 ± 8.8	8.9 ± 9.6	7.9 ± 5.9	0.38
Diabetes duration ≥10 years	155 (75.2)	112 (74.7)	43 (76.8)	0.75
HbA1c, %	8.8 ± 2.2	8.8 ± 2.3	8.6 ± 1.8	0.45
Body mass index ≥30 kg/m^2^	81 (38.9)	53 (35.1)	28 (49.1)	0.08
Number of medications	7.8 ± 4.1	7.8 ± 4.3	8.0 ± 3.8	0.75
Insulin treatment (yes)	142 (68.3)	103 (68.2)	39 (68.4)	0.98
Diabetes care units				<0.001
Full-time inpatient unit 1	53 (25.5)	34 (22.5)	19 (33.3)	
Weekday hospitalization unit	76 (36.5)	42 (27.8)	34 (59.6)	
Full-time inpatient unit 2	79 (38.0)	75 (49.7)	4 (7.0)	
Admission reasons				0.24
Imbalanced diabetes	135 (66.8)	97 (66.9)	38 (66.7)	
Diabetic foot	55 (27.2)	37 (25.5)	18 (31.6)	
Insulin pump installation	12 (5.9)	11 (7.6)	1 (1.7)	
Knowledge of mandatory vaccine (yes)	163 (78.4)	121 (80.1)	42 (73.7)	0.35
Knowledge of recommended vaccines				0.04
No	150 (72.1)	111 (73.5)	39 (68.4)	
Yes	5 (2.4)	1 (2.0)	4 (7.0)	
Incomplete	53 (25.5)	39 (25.8)	14 (24.6)	
Feelings about vaccination				0.01
For	100 (48.1)	64 (42.4)	36 (63.2)	
Against	19 (9.1)	17 (11.2)	2 (3.5)	
Mixed	53 (25.5)	45 (29.8)	8 (14.0)	
Without opinion	36 (17.3)	25 (16.6)	11 (19.3)	
Documentation in the medical record of the pharmacist’s assessment of the vaccination coverage on hospital admission (yes)	171 (82.2)	116 (76.8)	55 (96.5)	0.0009
Immunization coverage of Tdap (tetanus, diphtheria, and pertussis) vaccines at hospital admission				0.15
No	73 (35.1)	48 (31.8)	25 (43.9)	
Yes	91 (43.7)	72 (47.7)	19 (33.3)	
Unknown	44 (21.2)	31 (20.5)	13 (22.8)	
Immunization coverage of pneumococcal vaccines				0.17
No	174 (83.7)	122 (80.8)	52 (91.2)	
Yes	10 (4.8)	8 (5.3)	2 (3.5)	
Unknown	24 (11.5)	21 (13.9)	3 (5.3)	
Immunization coverage of influenza vaccines				0.22
No	118 (56.7)	90 (59.6)	28 (49.1)	
Yes	88 (42.3)	60 (39.7)	28 (49.1)	
Unknown	2 (1.0)	1 (0.7)	1 (1.8)	
Data are the mean ± SD, or n (%); HbA1c: hemoglobin A1c.				

**Table 2:** Immunization coverage at admission and discharge.

	Immunization Coverage at Admission			Immunization Coverage at Discharge		
	Total Population	Unit 2	Unit 1 and Weekday Unit	Total Population	Unit 2	Unit 1 and Weekday Unit
n	222	86 (38.7)	136 (61.3)	222	86 (38.7)	136 (61.3)
Tdap vaccines						
No	73 (32.9)	23 (26.7)	50 (36.8)	52 (23.4)	22 (25.6)	30 (22.0)
Yes	105 (47.3)	44 (51.2)	61 (44.8)	133 (59.9)	46 (53.5)	87 (64.0)
Unknown	44 (19.8)	19 (22.1)	25 (18.4)	37 (16.7)	18 (2039)	19 (14.0)
Pneumococcal vaccines						
No	174 (78.4)	65 (75.6)	109 (80.1)	132 (59.4)	64 (74.4)	68 (50.0)
Yes	24 (10.8)	10 (11.6)	14 (10.3)	69 (31.1)	11 (12.8)	58 (42.6)
Unknown	24 (10.8)	11 (12.8)	13 (9.6)	21 (9.5)	11 (12.8)	10 (7.4)
Influenza vaccines						
No	118 (53.2)	50 (58.1)	68 (50.0)	109 (49.1)	49 (57.0)	60 (44.1)
Yes	102 (45.9)	35 (40.7)	67 (49.3)	111 (50.0)	36 (41.9)	75 (55.2)
Unknown	2 (0.9)	1 (1.2)	1 (0.7)	2 (0.9)	1 (1.1)	1 (0.7)
Data are n (%). Tdap, tetanus, diphtheria, pertussis						

**Table 3:** Knowledge and feelings about vaccines.

	Total
n	222
Knowledge of mandatory vaccine (yes)	171 (77.0)
Knowledge of recommended vaccines	
No	155 (69.8)
Yes	8 (3.6)
Incomplete	59 (26.6)
Feelings about vaccination	
For	113 (50.9)
Against	19 (8.6)
Mixed	54 (24.3)
Without opinion	36 (16.2)
Reasons for patients against and with mixed feelings about vaccines (*n* = 63/73)	
Vaccines are not very efficient	17 (27.0)
Side effects of vaccines	19 (30.2)
Vaccination only if mandatory	18 (28.5)
Fear of vaccines	7 (11.1)
Others	2 (3.2)
Possession of a vaccination record booklet	59 (26.6%)
Data are n (%)	

**Table 4:** Factors associated with at least one vaccination during hospitalization: results of univariate and multivariable analyses.

Characteristics	Univariate Analysis Odds Ratio 95% CI	*p*-Value	Multivariate Analysis Odds Ratio 95% CI	*p*-Value
Sex, female (vs. male)	1.57 (0.85–2.90)	0.15	2.64 (1.05–6.64)	0.04
Diabetes care units		<0.0001		<0.0001
Full-time inpatient unit 1 (vs. full-time inpatient unit 2)	10.48 (3.31–33.14)		9.15 (2.33–35.97)	
Weekday hospitalization unit (vs. full-time inpatient unit 2)	15.18 (5.04–45.71)		22.62 (6.26–81.74)	
Admission reasons		0.24		0.13
Diabetic foot (vs. imbalanced diabetes)	1.24 (0.63–2.44)		3.42 (0.93–12.59)	
Insulin pump installation (vs. imbalanced diabetes)	0.23 (0.03–1.86)		0.30 (0.02–4.54)	
Type 2 diabetes (vs. type 1 diabetes)	1.48 (0.74–2.95)	0.31	0.59 (0.21–1.66)	0.13
Feelings about vaccination		0.02		0.0001
Against (vs. for)	0.21 (0.05–0.96)		0.08 (0.01–0.42)	
Mixed (vs. for)	0.32 (0.13–0.74)		0.18 (0.06–0.51)	
Without opinion (vs. for)	0.78 (0.35–1.77)		0.54 (0.18–1.58)	
Immunization coverage of Tdap (Tetanus, diphtheria, and pertussis) vaccines at hospital admission		0.16		0.21
Yes (vs. no)	0.51 (0.25–1.02)		0.48 (0.19–1.21)	
Unknown (vs. no)	0.81 (0.36–1.81)		1.12 (0.36–3.46)	
Immunization coverage of pneumococcal vaccines		0.20		0.03
Yes (vs. no)	0.59 (0.12–2.86)		0.27 (0.03–2.16)	
Unknown (vs. no)	0.34 (0.10–1.17)		0.1 (0.03–0.65)	
Immunization coverage of influenza vaccines yes (vs. no)	1.46 (0.79–2.70)	0.22		
Documentation in the medical record of the pharmacist’s assessment of the vaccination coverage on hospital admission (vs. no)	8.30 (1.93–35.74)	<0.001	5.14 (1.02–25.95)	0.04
Knowledge of recommended vaccines		0.10		
Yes (vs. no)	11.38 (1.24–104.94)			
Incomplete (vs. no)	1.02 (0.50–2.08)			
Body mass index ≥30 kg/m^2^ (vs. <30 kg/m^2^)	1.78 (0.96–3.31)	0.07		
all independent variables with a *p*-value < 0.25 in the bivariate analysis and one variable of interest (type of diabetes) were simultaneously introduced in the models				

## Data Availability

The data presented in this study are available on request from the corresponding author. The data are not publicly available due ethical restrictions.

## Supplementary Materials

The following supporting information can be downloaded at: https://www.mdpi.com/article/10.3390/medicina58020219/s1, Table S1: Characteristics of population at hospital admission according to coverage for Tdap (tetanus, diphtheria, pertussis), influenza, and pneumococcal vaccines.
