# Supplementary material for: Impact of Hospitalization in an Endocrinology Department on Vaccination Coverage in People Living with Diabetes: A Real-Life Study
Source: Medicina (Kaunas). 2022 Feb 1;58(2):219. doi: 10.3390/medicina58020219 (PMC8879927; doi:10.3390/medicina58020219)
Supplement: Supplementary file 1 [file medicina-58-00219-s001.zip › medicina-1537357-supplementary.pdf]

Supplementary Table S1. Characteristics of population at hospital admission according to coverage for Tdap (tetanus, diphtheria, pertussis), influenza and pneumococcal vaccines.

	Total	Tdap up to date	Tdap not up to date	Tdap unknown	p	Pneumococcal up to date	Pneumococcal not up to date	Pneumococcal unknown	p	Influenza up to date	Influenza not up to date	Influenza unknown	p
n	222	105 (47.3)	73 (33.9)	44 (19.8%)		24 (10.8%)	174 (78.4%)	24 (10.8%)		102 (45.9%)	118 (53.2%)	2 (0.9%)	
Age, years	59.4 ±15.2	57.4 ±16.0	61.4 ±13.6	60.9 ±15.8	0.18	59.5 ±18.6	59.7 ±14.2	56.8 ±19.2	0.68	63.9 ±13.0	55.5 (15.8)	56.50 ±37.5	0.0002
Sex male	131 (59.0)	72 (68.6)	35 (47.9)	24 (54.5)	0.02	16 (66.7)	105 (60.3)	10 (41.7)	0.16	72 (70.6)	59 (50.0)	0	0.001
Type 2 diabetes	152 (68.5)	70 (66.7)	53 (72.6)	29 (65.9)		12 (50.0)	126 (72.4)	14 (58.3)		67 (65.7)	84 (71.2)	1 (50.0)	0.45
Diabetes duration ≥10 years	165 (74.3)	81 (77.9)	48 (66.7)	36 (81.8)	0.12	18 (75.0)	128 (74.4)	19 (79.2)	0.88	88 (87.1)	77 (65.8)	0	<0.0001
Admission reasons					0.03				0.66				0.003
Imbalanced diabetes	140 (65.4)	55 (55.0)	53 (72.6)	32 (74.4)		13 (59.1)	113 (67.3)	14 (58.3)		55 (57.3)	85 (73.3)	0	
Diabetic foot	59 (27.6)	38 (38.0)	13 (17.8)	8 (18.6)		6 (27.3)	45 (26.8)	8 (33.3)		30 (31.2)	28 (24.1)	1 (50.0)	
Insulin pump installation	15 (7.0)	7 (7.0)	5 (6.9)	3 (7.0)		3 (13.6)	10 (5.9)	2 (8.4)		11 (11.5)	3 (2.6)	1 (50.0)	
Length of stay, mean, day	8.8 ±9.1	10.5 ±11.1	7.4 ±3.7	7.1 ±8.8	0.03	9.3 ±10.5	8.7 ±8.6	9.2 ±11.2	0.94	10.1 ±12.0	7.8 ±5.3	7.5 ±4.9	0.17
Number of medications	7.9 ±4.3	8.1 ±4.4	7.8 ±4.0	7.7 ±4.3	0.80	9.3 ±5.4	7.7 ±4.0	8.0 ±4.3	0.22	9.1 ±4.2	6.9 ±4.0	6.0 ±2.8	0.0003
Insulin treatment (yes)	152 (68.5)	68 (64.8)	50 (68.5)	34 (77.3)	0.32	18 (75.0)	115 (66.1)	19 (79.2)	0.33	77 (75.5)	73 (61.9)	2 (100.0)	0.06
HbA1c, %	8.7 ±2.2	8.6 ±2.1	8.7 ±2.3	9.1 ±2.1	0.51	7.9 ±1.4	8.9 ±4.5	8.5 ±2.1	0.11	8.5 ±1.8	8.9 ±2.4	7.9 ±1.4	0.23
BMI ≥30 kg/m^2	85 (38.3)	36 (34.3)	33 (45.2)	16 (36.4)		9 (37.5)	68 (39.1)	8 (33.3)		42 (41.2)	42 (35.6)	1 (50.0)	0.78

Data are mean ±SD, or n (%); BMI, body mass index, HbA1c, hemoglobin A1c.
